# A Community-Based Cross-Sectional Study to Assess the Prevalence of Undiagnosed Diabetes Mellitus in Vijayapura City

**DOI:** 10.7759/cureus.83050

**Published:** 2025-04-26

**Authors:** Sanketh C M, Vijaykumar G Warad, Anuja M Kadagud, Rahul Biradar

**Affiliations:** 1 General Medicine, Shri B. M. Patil Medical College Hospital and Research Centre, Bijapur Lingayat District Education (BLDE) (Deemed to be University), Vijayapura, IND

**Keywords:** diabetes, diabetes mellitus, diabetes mellitus type 2, diabetes screening, india, karnataka, prevalence study, symptoms of diabetes, undiagnosed diabetes mellitus, vijayapura

## Abstract

Background

Undiagnosed diabetes mellitus (UDM), a chronic hyperglycemic disorder, presents a growing global health challenge with significant morbidity and mortality risks stemming from delayed diagnosis and subsequent complications. Limited awareness, slow disease progression, and healthcare system limitations contribute to a substantial proportion of UDM, particularly in India, where the high economic burden of managing diabetes complications underscores the need for accurate UDM estimation to inform effective public health policies.

Objective

The primary objective of this community-based cross-sectional epidemiological study was to ascertain the prevalence of UDM within the urban populace aged 35 years and above in Vijayapura, Karnataka, India.

Methods

Conducted from May 2023 to December 2024 in urban Vijayapura, a sample of 1195 participants, stratified equally across six randomly selected geographical units within the city, underwent screening for UDM.

Results

The study determined a 5.61% prevalence of UDM through the identification of 67 new cases of diabetes mellitus among the screened urban residents.

Conclusion

This prevalence highlights the imperative for targeted screening and public health education programs to address the burden of UDM in this urban population aged 35 years and above.

## Introduction

Diabetes mellitus (DM) is a chronic metabolic disorder characterized by hyperglycemia resulting from absolute or relative deficiencies in insulin secretion and/or action. The global prevalence of diabetes in adults over 18 years of age rose from 180 million (4.7%) to 451 million (8.8%) between 1980 and 2017 [1-3]. DM was the direct cause of 4.0 million deaths in 2015, with half of them occurring in individuals under the age of 70 [1]. The World Health Organization (WHO) projects DM to be the seventh leading cause of mortality by 2030. Global estimates suggest that approximately 628.6 million individuals will be living with diabetes by the year 2045 [4].

Delayed diagnosis of DM is frequently a consequence of limitations within healthcare systems, inadequate public and professional awareness regarding the condition, and the often insidious onset or gradual progression of the disease. This protracted period of undiagnosed diabetes mellitus (UDM) exposes individuals to sustained hyperglycemia, leading to the development of significant and irreversible micro-vascular (peripheral vascular disease, neuropathy, nephropathy, retinopathy) and macro-vascular (coronary artery disease, stroke) sequelae [5,6]. UDM is associated with a significantly elevated mortality risk, ranging from 1.5 to 3.0 times that of individuals with normoglycemia. Notably, the mortality risk associated with UDM has been observed to be comparable to that of individuals with diagnosed DM [7,8].

The prevalence of DM is rising at a higher rate in low and lower-middle-income countries. According to International Diabetes Federation (IDF) global estimates of the prevalence of diabetes for 2011 and 2030, the greatest increase is expected in low-income countries (92%), followed by lower-middle-income countries like India (57%), upper-middle-income countries (46%), and finally, higher-income countries (25%) [9]. There are 77 million diabetics in India, and 43.9 million (57%) of them remain undiagnosed [10]. According to the India state-level disease burden initiative diabetes study collaborators, India's diabetes cases rose from 26 million in 1990 to 65 million in 2016, with adult prevalence increasing from 5.5% to 7.7%. State-level prevalence was highest in Tamil Nadu, followed by Karnataka, Kerala, Delhi, Punjab, and Goa [11].

Diabetes-related medical expenses have a significant financial impact on people, health systems, and governments in addition to the health burden; in 2013, it was projected that global health expenditures totaled at least 548.5 billion USD [12]. This estimate may be significantly impacted by the expense of UDM. The expense of diagnosing and treating DM is high, but it is greatly surpassed by the expense of treating complications from diabetes that may be avoided [12,13]. To comprehend the global and regional burden of UDM, its causes, and possible ramifications for practice and policy, it is critical to generate regional and global estimations of UDM.

Primary prevention of DM begins with targeted screening to identify high-risk individuals, including those with asymptomatic and undiagnosed disease. However, disparities in the availability and accessibility of diagnostic testing for diabetes contribute to its underdiagnosis. Therefore, this community-based cross-sectional study was conducted in urban Vijayapura, Karnataka, India, to assess the prevalence of UDM among adults aged 35 years and above and to highlight the need for enhanced screening strategies in this population.

## Materials and methods

Study design

This community-based cross-sectional study assessed the prevalence of UDM in individuals aged 35 years and above residing in urban Vijayapura between May 2023 and December 2024. Ethical approval was obtained from Shri B. M. Patil Medical College Hospital and Research Centre, Bijapur Lingayat District Education (BLDE) (Deemed to be University), and all participants provided written informed consent.

Study participants

The inclusion criteria were as follows: individuals residing in Vijayapura, aged 35 years or older, with no prior diagnosis of diabetes. Exclusion criteria included pregnant or lactating women and individuals taking medications known to induce hyperglycemia (e.g., corticosteroids).

Sampling method and data collection

A multi-stage sampling method was used. Six wards were randomly selected from Vijayapura's administrative units. Within each ward, households were systematically approached, and all eligible individuals were invited to participate, with up to two repeat visits for non-responders. A total of 1195 participants were screened, ensuring proportional representation across the selected wards.

Data collected included age, sex, random blood sugar (RBS) levels (using a regularly calibrated Accu-Chek Active Glucometer, Roche Diagnostics, Mannheim, Germany), and self-reported symptoms of hyperglycemia and diabetes (blurred vision, fatigue, frequent urination, excessive thirst, tingling/numbness, increased hunger, slow-healing sores/infections).

Data interpretation

**Figure 1:**
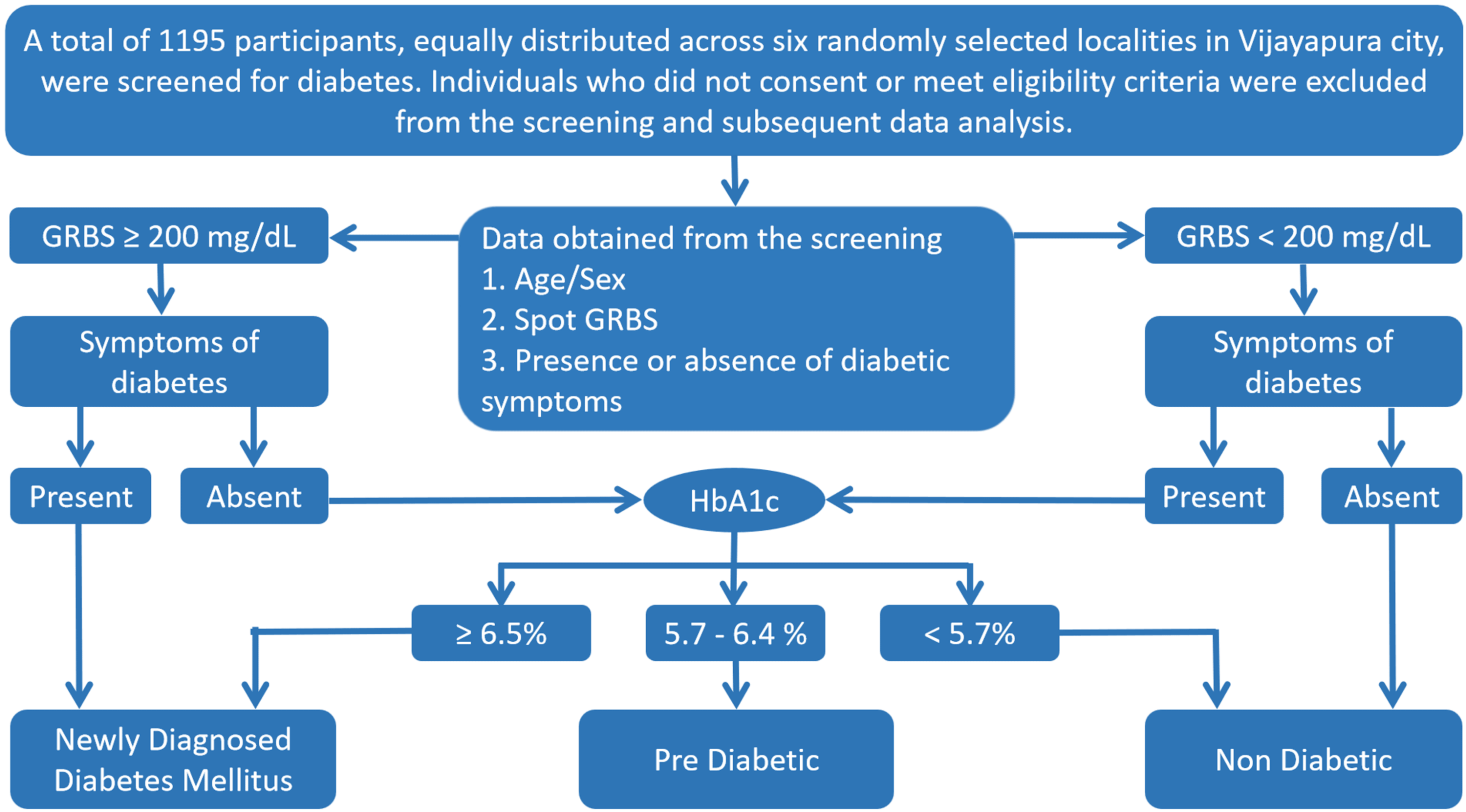
Flowchart representing methodology and data collection

Data interpretation followed the American Diabetes Association (ADA) guidelines [14]. Symptomatic individuals with RBS ≥ 200 mg/dL were classified as UDM. Asymptomatic individuals with RBS < 200 mg/dL were classified as non-diabetic initially. Symptomatic individuals with RBS < 200 mg/dL and asymptomatic individuals with RBS ≥ 200 mg/dL underwent HbA1c testing (at Central Research Laboratory, Shri B. M. Patil Medical College Hospital and Research Centre, BLDE (Deemed to be University) using high-performance liquid chromatography (HPLC)). Participants were categorized as UDM (HbA1c > 6.5%), pre-diabetic (HbA1c 5.7-6.4%), or non-diabetic (HbA1c < 5.7%) (Table 1 and Figure 1).

**Table 1:** Predetermined analytical frameworks used for data interpretation RBS, random blood sugar; UDM, undiagnosed diabetes mellitus

RBS (mg/dL)	Symptoms of diabetes	HbA1c (%)	Inference
≥ 200	Present	-	UDM
	Absent	≥ 6.5	UDM
		6.4-5.7	Pre-diabetic
		< 5.7	Non-diabetic
< 200	Absent	-	Non-diabetic
	Present	≥ 6.5	UDM
		6.4-5.7	Pre-diabetic
		< 5.7	Non-diabetic

Sample size

Based on a previous study indicating a 7.6% diabetes prevalence [15], a sample size was determined to estimate the prevalence of UDM in the urban population aged 35 years and above in Vijayapura with a 95% confidence level and a precision of 1.65%. A target sample size of 1195 participants was set to ensure adequate statistical power and account for potential non-response and ineligibility.

Statistical analysis

Data were entered in Microsoft Excel (Microsoft® Corp., Redmond, WA) and analyzed using SPSS (IBM SPSS Statistics for Windows, IBM Corp., Version 20, Armonk, NY). Descriptive statistics summarized participant characteristics and UDM prevalence. Group differences were assessed using t-tests (for normally distributed continuous data), Mann-Whitney U tests (for non-normal continuous data), and chi-square or Fisher's exact tests (for categorical data). ANOVA or Kruskal-Wallis H tests were used to compare more than two groups. A 95% confidence interval was used for all two-tailed statistical tests.

## Results

We screened 1195 participants aged 35 years and above from six randomly selected geographical areas in Vijayapura for UDM. Initial screening based on symptomatic presentation and an RBS level ≥ 200 mg/dL identified 60 participants with UDM. Subsequently, 120 additional symptomatic participants with RBS levels < 200 mg/dL underwent hemoglobin A1c (HbA1c) testing, revealing seven further cases of UDM and 24 individuals with pre-diabetes. Consequently, the overall study results showed 67 participants (5.61%) had UDM, 24 (2.01%) were pre-diabetic, and 1104 (92.38%) were non-diabetic (Table 2).

**Table 2:** Prevalence of UDM and pre-diabetics in Vijayapura UDM, undiagnosed diabetes mellitus

Category	N	Prevalence %	95% CI
UDM	67	5.61%	(4.3%, 6.9%)
Pre-diabetic	24	2.01%	(1.2%, 2.8%)
Non-diabetics	1104	92.38%	(91%, 94%)
Total participants screened	1195		

Among the 1195 participants screened for diabetes (599 females and 596 males), 35 females and 32 males had UDM. The gender-specific prevalence of UDM was slightly higher in females (2.93%; 95% CI: 2%, 3.9%) than in males (2.68%; 95% CI: 1.8%, 3.6%) (Table 3).

**Table 3:** Gender-wise prevalence of UDM in Vijayapura UDM, undiagnosed diabetes mellitus

Gender	Participants screened	UDM	Prevalence %	95% CI
Female	599	35	2.93%	(2%, 3.9%)
Male	596	32	2.68%	(1.8%, 3.6%)
Total	1195	67	5.61%	(4.3%, 6.9%)

The age-specific prevalence of UDM is stratified into six age groups: 35-44, 45-54, 55-64, 65-74, 75-85, and >85 years, and the number of participants screened varied across age groups (Table 4). The 45-54 year and 55-64 year age groups had the largest sample sizes (n = 287 each), and the >85 years age group the smallest (n = 60). The prevalence of UDM increased with age, reaching a peak of 6.97% (20 cases out of 287) in the 55-64 year age group, followed by a decline in older age groups, reaching 5% (three cases out of 60) in the >85 year age group. However, the decline in prevalence in the older age groups suggests a non-linear relationship across the entire age spectrum (Table 4).

**Table 4:** Age distribution of participants and the age-specific prevalence of UDM in Vijayapura UDM, undiagnosed diabetes mellitus

Age group	Participants screened (N)	UDM (n)	Prevalence %	95% CI
35-44 years	202	5	2.48%	(0.8%, 5.7%)
45-54 years	287	11	3.83%	(1.9%, 6.9%)
55-64 years	287	20	6.97%	(4.3%, 10.4%)
65-74 years	239	17	7.11%	(4.3%, 11.2%)
75-85 years	120	11	9.17%	(4.6%, 16.3%)
>85 years	60	3	5.00%	(1.0%, 14.0%)

**Figure 2:**
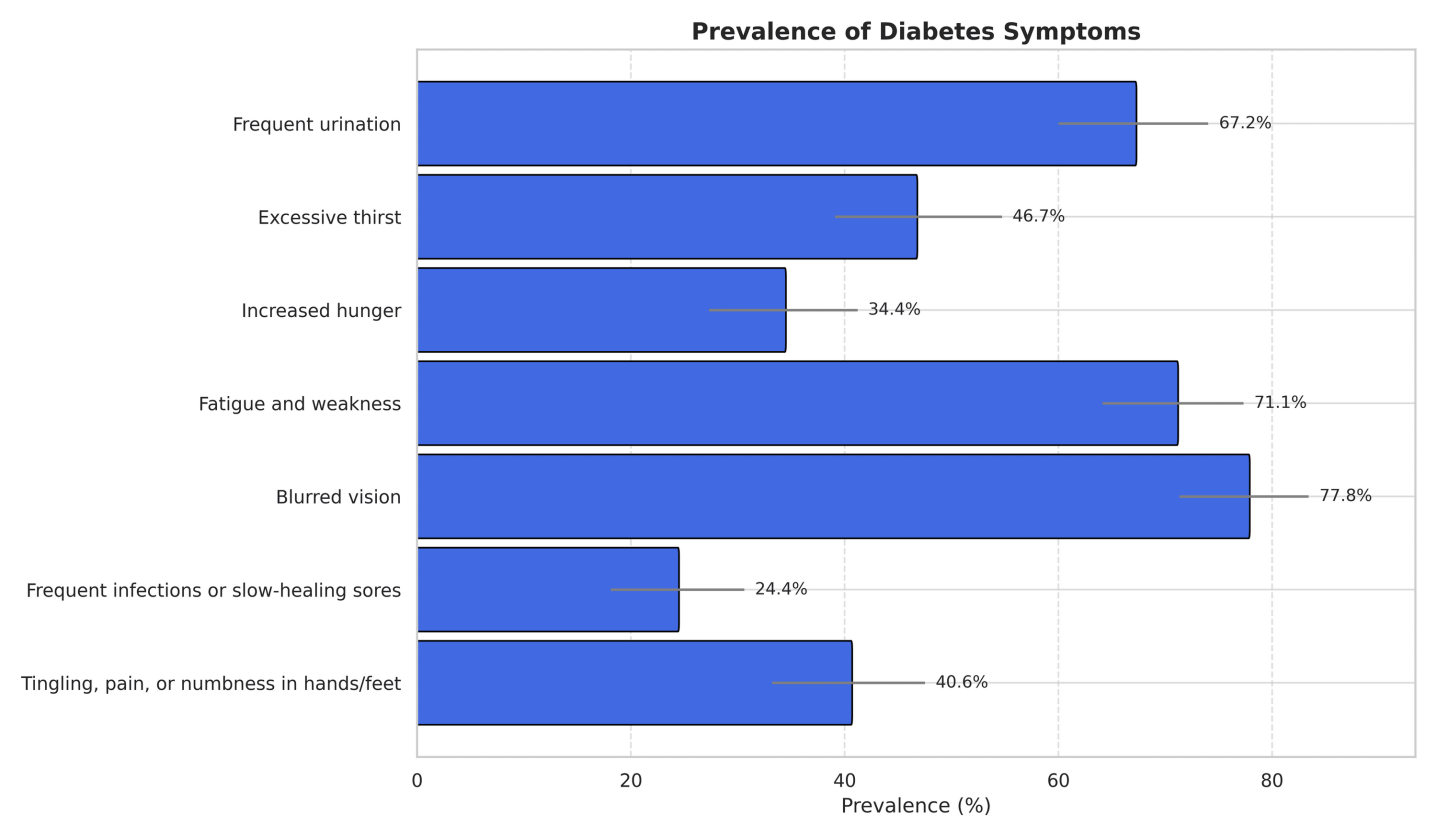
Distribution of symptoms of diabetes mellitus

Among 1195 participants screened, 180 (15.1%) reported having one or more symptoms of diabetes. The most common symptoms were blurred vision (77.8%, 95% CI: 71.3%, 83.4%), fatigue and weakness (71.1%, 95% CI: 64.1%, 77.3%), and frequent urination (67.2%, 95% CI: 60%, 74%). Other frequently reported symptoms included excessive thirst (46.7%, 95% CI: 39.1%, 54.7%), tingling, pain, or numbness in hands/feet (40.6%, 95% CI: 33.2%, 47.5%), and increased hunger (34.4%, 95% CI: 27.3%, 41.2%). Frequent infections or slow-healing sores were observed in 24.4% (95% CI: 18.1%, 30.6%) of individuals (Table 5 and Figure 2).

**Table 5:** Distribution of symptoms of diabetes mellitus among participants reporting one or more symptoms (N = 180)

Symptoms of diabetes	N	Prevalence %	95% CI
Frequent urination	121	67.2%	(60%, 74%)
Excessive thirst	84	46.7%	(39.1%, 54.7%)
Increased hunger	62	34.4%	(27.3%, 41.2%)
Fatigue and weakness	128	71.1%	(64.1%, 77.3%)
Blurred vision	140	77.8%	(71.3%, 83.4%)
Frequent infections or slow-healing sores	44	24.4%	(18.1%, 30.6%)
Tingling, pain, or numbness in hands/feet	73	40.6%	(33.2%, 47.5%)

## Discussion

The high rate of UDM in Vijayapura is a serious concern, particularly because a significant portion of these individuals presented with diabetic symptoms, yet remained unaware of their condition, likely due to limited knowledge about the disease. These findings are consistent with previous studies conducted in India and other countries.

For instance, a study by Pradeepa and Mohan estimated that 57% of adults with diabetes in India remain undiagnosed, corresponding to approximately 43.9 million individuals. The authors emphasized that many individuals are unaware of their diabetic status due to a lack of screening and asymptomatic progression in early stages [10]. International studies have also reported a high prevalence of UDM. A study in Northwest Ethiopia found a UDM prevalence of 10.2%, with risk factors including a lack of knowledge of symptoms of diabetes and family history [16]. This is higher than the 5.61% prevalence observed in the current study, likely due to differences in lifestyle, genetic predisposition, and access to healthcare.

In contrast, the national diabetes statistics report of the United States reported a 3.4% prevalence rate of UDM in the country [17]. Similarly, a study in China also reported a lower prevalence of UDM (4.4%) [18]. This lower prevalence is likely due to widespread access to healthcare services and early detection programs in these countries.

Our study showed that UDM prevalence in urban Vijayapura followed a non-linear trend with age, increasing to a peak in middle age and then decreasing in both younger and older age groups, with the highest proportion of UDM observed in the 55-64 year age group, followed by the 65-74 year age group. These findings align with previous studies indicating that diabetes prevalence increases with age. For instance, the National Non-communicable Disease Monitoring Survey conducted in India between 2017 and 2018 found that diabetes prevalence sharply rises after 30-49 years of age, with individuals aged 50-69 years having the highest burden of diabetes in India [19].

However, in contrast to our study, Sahadevan et al.'s analysis of National Family Health Survey (NFHS)-5 (2019-2021) data in individuals under 50 years yielded a 1.22% prevalence of UDM. This disparity is likely explained by the substantial age difference between their younger population and our older cohort [20].

Our study's results align with the community-based cross-sectional study conducted in Bahir Dar city of northwest Ethiopia, which found the highest prevalence of UDM in the 55-64 age group, at 21.9% [16]. On the other hand, in China, a national diabetes screening program identified a more even age distribution across younger and older adults, suggesting that earlier detection strategies may help mitigate age-related diabetes progression [21].

The results of our study underscore the urgent need for the adoption of widespread diabetes screening and comprehensive public awareness and health education strategies in India, particularly targeting both urban and rural regions with limited healthcare resources. Given the increasing burden of DM in India, the successful implementation of these interventions, alongside improved healthcare accessibility, is critical for decreasing the prevalence of UDM.

This study possesses notable strengths in its methodology, employing a large, representative sample achieved through stratified random sampling and community-based screening. The implementation of symptom-driven HbA1c follow-up for symptomatic individuals exhibiting normal RBS values enhanced the precision of diabetes detection by minimizing false negatives. Nevertheless, several limitations warrant consideration. The exclusion of participants under 35 years of age, the reliance on symptom-based HbA1c testing, and the absence of fasting blood sugar (FBS) screening suggest that the estimated prevalence of UDM within Vijayapura may not fully reflect the true prevalence. These limitations, arising from pragmatic considerations of cost and participant compliance, potentially resulted in reduced sensitivity for identifying UDM cases, a possible underestimation of overall prevalence, particularly concerning asymptomatic presentations, and a likely under-representation of type 1 diabetes within the study population.

## Conclusions

Our study reveals a substantial prevalence of UDM among individuals aged 35 years and above in Vijayapura. The persistence of UDM, even in participants with overt symptoms, highlights a critical knowledge deficit. Middle age represents a period of peak prevalence, underscoring the necessity for early detection through targeted community screening and public health education to mitigate diabetes-related morbidity.
